# Combating Climate Change in the Kenyan Tea Industry

**DOI:** 10.3389/fpls.2020.00339

**Published:** 2020-03-25

**Authors:** Chalo Richard Muoki, Tony Kipkoech Maritim, Wyclife Agumba Oluoch, Samson Machohi Kamunya, John Kipkoech Bore

**Affiliations:** ^1^Crop Improvement and Management Programme, Kenya Agricultural and Livestock Research Organization, Tea Research Institute, Kericho, Kenya; ^2^Sustainable Ecosystem Management and Conservation Programme, Kenya Agricultural and Livestock Research Organization, Tea Research Institute, Kericho, Kenya

**Keywords:** *Camellia*, modeling, breeding, molecular, physiology

## Abstract

Climate change triggered by global warming poses a major threat to agricultural systems globally. This phenomenon is characterized by emergence of pests and diseases, extreme weather events, such as prolonged drought, high intensity rains, hailstones and frosts, which are becoming more frequent ultimately impacting negatively to agricultural production including rain-fed tea cultivation. Kenya is predominantly an agricultural based economy, with the tea sector generating about 26% of the total export earnings and about 4% gross domestic product (GDP). In the recent years, however, the country has witnessed unstable trends in tea production associated with climate driven stresses. Toward mitigation and adaptation of climate change, multiple approaches for impact assessment, intensity prediction and adaptation have been advanced in the Kenyan tea sub-sector. Further, pressure on tea breeders to release improved climate-compatible cultivars for the rapidly deteriorating environment has resulted in the adoption of a multi-targeted approach seeking to understand the complex molecular regulatory networks associated with biotic and abiotic stresses adaptation and tolerance in tea. Genetic modeling, a powerful tool that assists in breeding process, has also been adopted for selection of tea cultivars for optimal performance under varying climatic conditions. A range of physiological and biochemical responses known to counteract the effects of environmental stresses in most plants that include lowering the rates of cellular growth and net photosynthesis, stomatal closure, and the accumulation of organic solutes such as sugar alcohols, or osmolytes have been used to support breeding programs through screening of new tea cultivars suitable for changing environment. This review describes simulation models combined with high resolution climate change scenarios required to quantify the relative importance of climate change on tea production. In addition, both biodiversity and ecosystem based approaches are described as a part of an overall adaptation strategy to mitigate adverse effects of climate change on tea in Kenya and gaps highlighted for urgent investigations.

## Introduction

Human activity is driving significant changes in global and regional climate systems through enhanced greenhouse effects (Intergovernmental Panel on Climate Change, 2014). Global climate models predict that these changes will alter both mean climate parameters and the frequency and magnitude of extreme meteorological events that may include heat waves, severe storm events and drought (Semenov and Halford, 2009). Such changes may have significant destabilizing effects, decoupling existing relationships between species, altering species distributions and challenging current management regimes. Understanding and predicting the impacts of climate change on agricultural ecosystem processes is thus critical.

Tea, *Camellia sinensis* (L.) O Kuntze, originated from areas of monsoon climates with a warm, wet summer and a cool, dry (or less wet) winter. However, with dispersal the plant is now grown in conditions which range from Mediterranean-type climates to the hot humid tropics (Carr, 1972). It is an economically important crop, extensively consumed as non-alcoholic beverage across the globe. It is profoundly known for its taste, flavor, aroma and medicinal properties attributed to rich beneficial secondary metabolites (Jin et al., 2018). As a perennial plant, tea encounters a large number of environmental stresses throughout its life span. The minimum annual rainfall generally considered sufficient for the successful cultivation of tea varies between 1150 and 1400 mm per year (Carr, 1972). In most tea growing areas, well distributed rainfall (150 mm per month) ensures continuous crop production. A positive correlation between the integrated measure of air temperature and the rate of shoot extension has been reported. Minimum air temperature required to support shoot growth is about 13–14°C, with optimum range of 18–30°C. Excessive daytime maximum temperature beyond 30°C is known to restrict shoot growth rate, whereas freezing night temperature followed by a rapid rise in day temperature (night frost) leads to leaf scorching (Eden, 1965). Relatively higher day temperatures as compared to night temperature, leaf temperature below 35°C and soil temperature between 20 and 25°C are considered optimum for tea growth (Carr, 2010a,b). Also long photoperiods are essential for maximum yield (Barua, 1969). Studies across the tea growing regions have revealed that the weather is becoming more erratic and less predictable: more hot days, reduced number of rainy days and discernible decline in the annual hours of sunshine (Esham and Garforth, 2013; Bore and Nyabundi, 2016; Han et al., 2016; Ochieng et al., 2016; Papalexiou et al., 2018).

**FIGURE 1:**
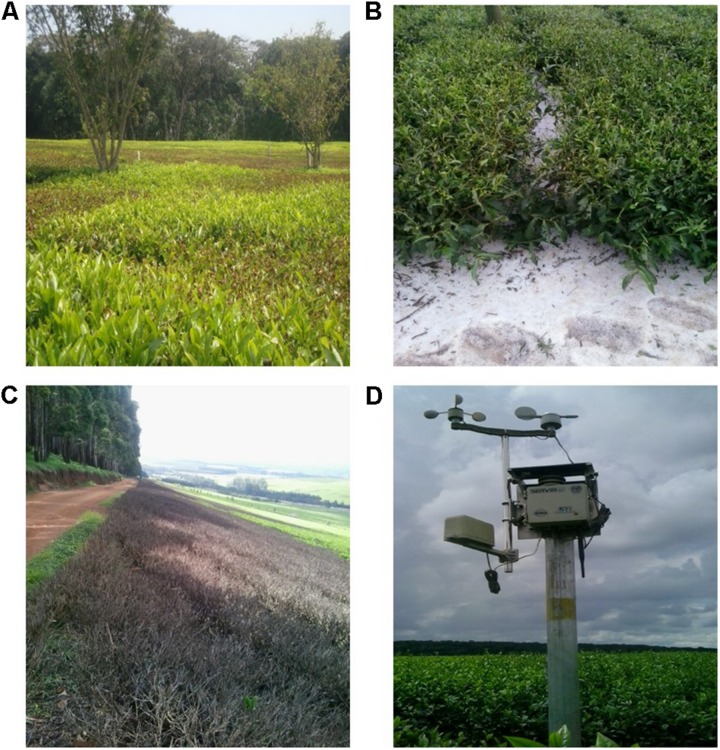
**(A)** Effect of frost in tea plantation. **(B)** Severe hailstone damage resulting in suspension of tea plucking for several months. **(C)** Effect of planting eucalyptus trees near tea fields. **(D)** Wireless sensor network (WSN) that assist in timely weather data deployment.

Kenya is predominantly an agricultural based economy. Tea was reportedly introduced in the country by the Caine brothers who imported dark-leafed “Manipuri” hybrid seeds from Assam in 1904 and 1905 to establish a plantation at Limuru, Central Kenya (Matheson, 1950). In 1912, Chinary (var. *sinensis*) seeds were imported from Sri-Lanka to establish a plantation of tea with high quality and yield (Matheson, 1950). Planting expanded rapidly from 1924 following advice on the use of quality seeds from the light colored leaf Assam or Manipuri types for drought resistance (Greenway, 1945). In the year 2018, Kenya produced 493 Million Kg earning the country over Kshs. 140 Billion in foreign exchange. This represents about 26% of the total export earnings, and about 4% gross domestic product (GDP) (Wachira, 2002; Kamunya et al., 2012; Azapagic et al., 2016). The country has more than 232,742 hectares of tea (International Tea Committee, 2018) spread in 18 counties and due to the low level of mechanization involved in cultivation, it offers direct and/or indirect employment to over 10% of the population. Further, because the industry is largely rural based, it contributes to both the local rural economies and reduces rural-urban migration (Wachira, 2002). Sustainability of the industry is thus crucial to the country’s socio-economic well-being and development. Being a rainfed plantation crop in Kenya, tea depends greatly on weather for optimal growth. The plant is grown in high altitude areas East and West of the Great Rift Valley, between 1400 and 2700 m amsl, where rainfall ranges between 1800 and 2500 mm annually. Evidence suggests a negative impact of global warming on production and quality of tea, especially with regards to temperature rise, unpredictable rainfall trends and increasing frequency of extreme weather events such as hail storms, drought and frost (Figure 1; Boehm et al., 2016; Ahmed et al., 2018, 2019). Studies have documented that stress, especially drought, account for 14–20% loss in yield and 6–19% plant mortality (Ng’etich et al., 2001; Cheruiyot et al., 2007). Multiple environmental parameters are known to impact tea quality, although the directionality and magnitude is not clear likely due to variations in various factors such as cultivar, environment and management conditions (Ahmed et al., 2019). Under such circumstances, tea production is vulnerable to the predicted climate change effects and, subsequently, greater economic, social, and environmental problems. There is need for scientific and community-based adaptation and mitigation strategies. Adoption of a multi-targeted approaches that seek to understand the complex physiological, biochemical and molecular regulatory networks associated with stress response will ensure sustainability of the tea sector. These necessitate intense research to improve tea production under diverse stress conditions.

## Impact of Climate Change on Tea Production

With climate change, it is expected that the main tea growing areas will experience an increase in the length of dry seasons per year, warmer temperatures and/or extreme rainfall intensity (Wijeratne, 1996; Trejo-Calzada and O’Connell, 2005; Eitzinger et al., 2011). Climate data collected at KALRO-TRI for over 58 years, indicate an annual temperature rise of 0.016°C per year while annual rainfall decreased by 4.82 mm per year over the same period (Cheserek et al., 2015). This has led to continued increase in soil water deficit (SWD) over time. On an annual basis, a large SWD, especially in January, February and March is reported leading to significant oscillations in tea production annually (Bore, 2008).

**FIGURE 2:**
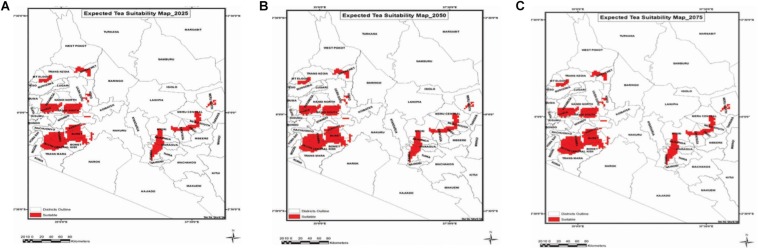
Projected suitable areas for tea growing in Kenya. **(A)** 2025, **(B)** 2050, and **(C)** 2075. Source: Adapted from Bore and Nyabundi (2016).

Simulation models provide the best known approach for integrating our understanding of complex plant processes that are influenced by weather and other environmental factors. Models are useful in guiding the direction of fundamental research by providing quantitative predictions and highlighting gaps in our knowledge (Semenov and Halford, 2009). Their use in assessing the impact of climate change and identifying potential future threats in plant sciences has been extensively reported (Sinclair and Muchow, 2001; Porter and Semenov, 2005; Passioura, 2007). Simulation models combined with high resolution climate change scenarios have been used to quantify the regions that could be suitable for economic production of tea in Kenya by the year 2075 (Figure 2; Bore and Nyabundi, 2016). Maps generated in a GIS environment using climate data from the Kenya Meteorological Services predicted that the mean air temperature for the region would increase by about 2% by 2025 and by 11% by 2075, if no action is taken. Distribution of areas suitable for tea cultivation within the current growing areas in Kenya will decrease. This is attributed to rainfall distribution and not amounts of rainfall received. The rise in mean air temperatures beyond the threshold of 23.5°C might also occur. Further, suitability of tea growing areas is expected to decline by 22.5% by the year 2075 while a suitability increase of 8% is expected by 2025. In order to boost the adaptation and performance of tea, a key strategy would be first to understand the mechanisms involved in stress tolerance, and then use appropriate tools and breed for stress tolerance.

Considering the established positive influence of temperature on tea production, it is imperative to conduct the economics of supplying water to tea fields during drought to reap from the enterprise (Cheserek et al., 2015). The irrigation or fertigation possibilities in tea had been documented in earlier experiments conducted in Sri Lanka (Wijeratne et al., 2007), India (Panda et al., 2003) and East Africa (Carr, 1972, 2010a,b). Timing of irrigation in tea fields has also been determined with inherent benefits (Carr, 1972; Othieno, 1978). This paves way for simulations into the projected temperature scenarios to reveal the potential yield levels. The divide between the cooler and already warm places could also support regional specific temperature thresholds for the crop hence support breeding activities for site specific cultivars with better heat stress tolerance.

Recent works were driven principally by the emergence of improved cultivars which had poor rooting system hence subject to water stress problems. Relations between young grafted teas with water stress had been done (Bore et al., 2010). How plants recover from drought event has also been determined (Muoki et al., 2012) as well as response of composite tea to progressive drought (Bore et al., 2010). A remarkable breakthrough in tea plant and drought relations was established revealing the threshold moisture content below which tea plant succumbs (Cheruiyot et al., 2008). Deeper understanding of the relationships could be achieved via controlled carbon dioxide (CO_2_) enrichment experiments such as Free Air Carbon Enrichment (FACE). There is need for development of cultivars which are not only tolerant to heat stress but equally adaptable to higher CO_2_ levels in the atmosphere (Wijeratne et al., 2007). A significant increase in concentrations of total catechins, other polyphenols and amino acids have been reported elsewhere with elevated carbon dioxide, while caffeine levels decrease (Li et al., 2017). Studies on carbon enrichment in tea cultivars grown in Kenya have, however, not been quantified though clear increase in temperature as CO_2_ rises has been established.

## Response of Tea to Climate Change

Plant responses to stress are dynamic and complex. This is often manifested by its physiological and biochemical reactions, which can provide a basis for screening for and selection of individual varieties and germplasm resistant to stress factors. Such responses include stomatal closure, repression of cell growth and photosynthesis, accumulation of organic osmolytes, and activation of respiration (Muoki et al., 2012; Maritim et al., 2015). Several studies have reported the effects of stress on critical components present in tea and corresponding synthetic genes. The present section focuses on providing an overview of the physiological, biochemical and molecular mechanisms of stress response and tolerance in tea (Table 1). This will provide theoretical knowledge for development of climate-resilient tea cultivars as the parameters described can be used as stress index for screening and clonal selection.

**TABLE 1:** An overview of various breeding strategies employed to improve tolerance of tea to adverse environmental factors in Kenya.

Techniques/approaches	Target trait	References
***Physiological characterization***		
Relative water content	Drought tolerance	Maritim et al., 2015; Cheruiyot et al., 2007
Shoot water potential	Drought tolerance	Maritim et al., 2015; Cheruiyot et al., 2007
Gas exchange measurement	Drought tolerance	Maritim et al., 2015; Cheruiyot et al., 2007
Genotype (G) × Environment (E) interaction	Yield	Wachira et al., 2002
***Biochemical characterization***		
Total Polyphenols	Drought tolerance	Cheruiyot et al., 2007
Amino acids (Proline)	Drought tolerance	Maritim et al., 2015
Amines (Glycinebetaine)	Drought tolerance	Maritim et al., 2015
Epicatechin and Epigallocatechin	Drought tolerance	Cheruiyot et al., 2008
***Physiological:Biochemical characterization***		
Short-time Withering Assessment of Probability for Drought Tolerance	Drought tolerance	Nyarukowa et al., 2016
Combining ability	Drought tolerance, Quality and Yield	Kamunya et al., 2009
***Molecular approaches***		
Linkage analysis and QTL mapping (RAPD, AFLP and SSR)	Yield	Kamunya et al., 2010
Linkage analysis and QTL mapping (DArT)	Drought tolerance and quality	Koech et al., 2018
Bulked segregant analysis (BSA)	Yield	Kamunya et al., 2010
Genomics	Drought tolerance and black tea quality	Koech et al., 2019
Suppression subtractive hybridization	Drought tolerance	Muoki et al., 2012
Transcriptomics	Drought tolerance	Maritim et al., 2016

### Physiological Responses

Climate change induced stresses affect plant systems such as photosynthesis, respiration and water retaining capacity. Tea plants exhibit C_3_ mechanism of photosynthesis, a key process affected by water deficits, via decreased CO_2_ diffusion to the chloroplast leading to metabolic constraints (Tezara et al., 2002; De Costa et al., 2007; Pinheiro and Chaves, 2011). Relative impact of such limitations varies with the occurrence and intensity of stress. Rate of photosynthesis in tea increases up to an illuminance (photon flux density) of about 1000 μmol m^–2^ s^–1^ and then remains relatively constant (Smith et al., 1993), while the optimum leaf temperature for photosynthesis in tea is about 25–30°C (Smith et al., 1993; Mohotti and Lawlor, 2002; Barman et al., 2008). Under stress condition, the photosynthetic machinery of the tea plant are damaged, hence limiting the stomatal conductance of the leaves and eventually leading to a significant decline in net photosynthesis and respiration rate. Using drought resistant and susceptible tea cultivars, several studies have reported a significant difference in photosynthesis and respiration rate following reduction in soil moisture content (Netto et al., 2010; Lin et al., 2014; Maritim et al., 2015).

Tea has a critical xylem water potential value of −0.7 to −0.8 megapascal (MPa) in relation to potential SWD and saturation deficits of the air (Carr, 2010b). Previous studies have highlighted key physiological responses in relation to water deficit in tea (Cheruiyot et al., 2007; Maritim et al., 2015). Relative water content (RWC) is one of the most important measures of plant water status when plants are exposed to drought and heat stress. It reflects the degree of plants water status, retaining or regulation capacity (Anjum et al., 2011). RWC varies according to genotypes, with resistant genotypes maintaining higher RWC compared to susceptible ones (Maritim et al., 2015). Furthermore, a method for Short-time Withering Assessment of Probability for Drought Tolerance (SWAPDT) validated by targeted metabolomics for predicting the drought tolerance (DT) in tea was developed (Nyarukowa et al., 2016). The method relies on the percent RWC of tea leaves after 5 h under withering conditions. Based on metabolite profiles, drought tolerant tea cultivars differed from drought susceptible tea cultivars providing a basis for selection of new drought tolerant tea cultivars that may lead to improvement of crop productivity, amidst challenges imposed by drought due to climate change.

### Biochemical Responses

As water is being removed from the cell, osmotic potential is reduced due to the effect of solute concentration (Yamada et al., 2005). However, if during the course of cellular water loss solutes are actively accumulated, osmotic potential would be reduced beyond the rate dictated by the mere effect of concentration. These involve the accumulation of organic compounds such as amino acids (e.g., proline), quaternary and other amines (e.g., glycinebetaine and polyamines) and a variety of sugars and sugar alcohols (e.g., mannitol, trehalose, and galactinol). Proline is widely studied because of its considerable role in stabilizing sub-cellular structures, scavenging free radicals, and buffering cellular redox potential under stress conditions (Ashraf and Foolad, 2007). In tea, proline accumulation under stress is significantly correlated with stress tolerance, and its concentration has been shown to be higher in stress-tolerant than in stress-sensitive plants (Chakraborty et al., 2002; Maritim et al., 2015). However, its use as a drought index is cultivar dependent. Nevertheless, stresses beyond tolerance levels will induce oxidative damage due to intensive production of reactive oxygen species (ROS) (Smirnoff, 1993). Glycinebetaine has also been reported to increase under stress condition (Maritim et al., 2015). Furthermore, tolerant cultivars have been reported to maintain higher polyphenol content at low SWC suggesting that cultivars with more stable polyphenols are more tolerant to water stress (Cheruiyot et al., 2007). Phenolic compounds can thus be useful indicators of DT in tea and will hasten the development of better-adapted cultivars to water-stress environments.

### Genomic Responses

Rapid progress in molecular breeding in tea is attributable to advances in genomics technologies, especially DNA sequencing, leading to publication of two draft genomes (Xia et al., 2017; Wei et al., 2018). In Kenya, the approach has been integrated into tea improvement programs. Muoki et al. (2012) used subtracted cDNA libraries from irrigated and drought stressed plants of a tolerant cultivar to understand the molecular responses of tea to abiotic stresses, especially drought. With progressive drought, genes related to chaperones, cell rescue/defense and cellular transport categories exhibited an early up-regulation in tolerant as compared to the susceptible variety. Dysfunction of enzymes and proteins usually accompanies abiotic stresses. Plants induce the expression of chaperones to ensure protein stabilization and cellular homeostasis during stress (Wang et al., 2004). Maintenance of proteins in their functional conformations and prevention of aggregation of non-native proteins is particularly important for cell survival under stress (Muoki et al., 2012). Molecular chaperones function in the stabilization of proteins and membranes, and assist protein refolding under stress conditions (Wang et al., 2003). In addition, Maritim et al. (2016) reported a significant upregulation of drought-related genes such as heat shock proteins (HSP70), superoxide dismutase (SOD), gene catalase (CAT), ascorbate peroxidase (APX), calmodulin-like protein (Cam7) and galactinol synthase (Gols4) in drought tolerant as compared to drought sensitive tea cultivars. Further, three major enzymes, namely transferases, hydrolases and oxidoreductases are involved in flavonoid biosynthesis, alkaloid biosynthesis, ATPase family proteins related to abiotic/biotic stress response have been identified (Koech et al., 2019). However, plants have evolved various antioxidative systems to keep the levels of ROS under control (Mittler, 2002). ROS are capable of unrestricted oxidation of various cellular components and can damage cell membranes and macromolecules. Many abiotic stresses directly or indirectly affect the synthesis, concentration, metabolism, transport and storage of important carbohydrates in plants. Soluble sugars are known to act as potential signals interacting with light, nitrogen and abiotic stress to regulate plant growth and development (Cramer et al., 2011). Overall, the level of soluble sugars increases with progressive drought stress in tea, wherein drought tolerant cultivars maintained higher levels as compared to the susceptible cultivars (Damayanthi et al., 2010). This finding indicated the ability of the tolerant cultivars to withstand drought by osmotic adjustments. Data generated from these studies provide critical resource for development of markers that can be used for selection of climate resilient tea cultivars.

## Breeding and Selection

### Conventional Approach

**FIGURE 3:**
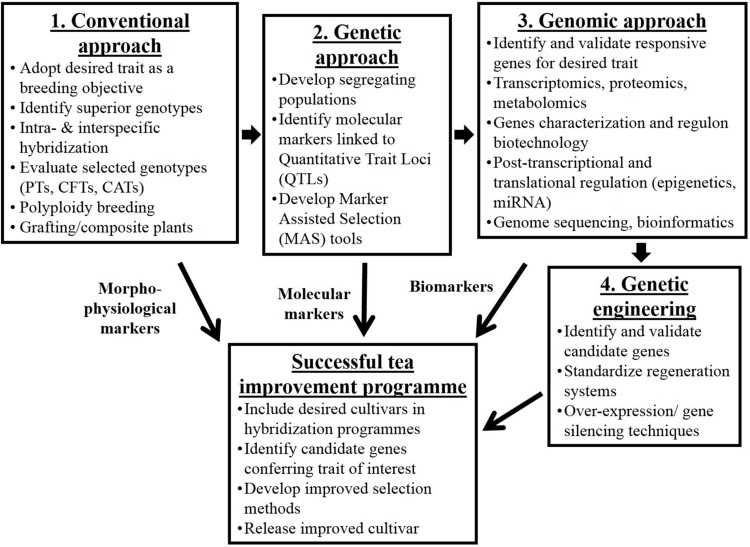
Schematic diagram on tea improvement strategies and techniques that have evolved over a period of time. Arrows in between boxes **1**, **2**, **3**, and **4** show the evolutionary time scale of the development of the strategies. CATs, clonal adaptability trials; CFTs, clonal field trials; MAS, marker assisted selection; PTs, progeny trials; QTLs, quantitative trait loci.

Sustainability and profitability of the tea industry depends primarily on the availability of desired planting materials. Most of the genetic improvement and the substantial increase in tea yields realized this far is brought about by conventional breeding through selection for hybrid vigor, though the process has continued to evolve over the years (Figure 3). Tea breeding essentially consists of four phases; generation of genetic variability, selection of useful genotypes and comparative tests to demonstrate the superiority of the selected genotypes. A fourth phase that involves exposing pre-released and promising clones to multiple sites (genotype-environment interaction) for stability and adaptability is always the final phase in plant improvement programs (Wachira et al., 2002; Kamunya et al., 2010). It is worthwhile to note that TRI has developed over 1,000 improved cultivars, out of which 58 cultivars have been selected for consistent superiority in yield and quality and released for commercial exploitation. Fourteen of these clones are capable of yielding between 5,000 and 8,000 kg of made tea per hectare per year. These yield levels are some of the highest in the world and are in the magnitude of three times the average yields of unimproved tea.

Approaches involving intravarietal and interspecific hybridizations have also been tapped as means of introducing desirable traits (Kamunya et al., 2012). The approach is facilitated by the availability of diverse genetic collection, standardized vegetative propagation procedures, continuous germplasm enrichment through material transfers between research institutions and the comparatively low operational costs involved. A remarkable achievement of conventional breeding was the transition from pioneer seedling tea plantations to the adoption of modern high-yielding vegetatively propagated cultivars. This led to a drastic increase in tea production globally in the mid-20th century. The technique reduced the juvenile period to as short as 6 months from the protracted 3 years for seed raised tea plants (Wachira, 2000). Given the financial considerations associated with clonal teas, farmers are now uprooting and replacing the old and diverse seedling tea plantations with a few improved clones. As clones represent instantly fixed genotypes, the practice means over-reliance on a limited number of cultivars, implying that on-farm diversity is minimizing and the risks posed by co-evolving challenges associated with climate change is increasing. Another emanating problem is that most tea breeding programs rely heavily on a few clonal parents as donors of desired genes, thereby manifesting the potential danger of mono-cropping (Wachira, 2002). For instance, 67% of released varieties in Kenya share the same female parent, cultivar TRFK 6/8 which is susceptible to root knot nematodes.

As water resources for agriculture become more limiting, the need to develop drought tolerant cultivars is increasingly gaining importance. The ability of plants to tolerate changes in extremes of abiotic stress conditions is a complex and coordinated response, involving hundreds of genes. These responses are also affected by interactions between the different environmental factors and the developmental stage of the plant. Breeding involves genetic alteration or modification of organisms through natural or human-imposed mutations or crosses. This process has continued to evolve in tea over the years. A foundation in conventional breeding has contributed significantly to tea improvement. This involves the identification of stress tolerant parents (Table 2), intra- or interspecific hybridization, establishment of progeny trials (PTs), clonal field trials (CFTs), and clonal adaptability trials (CATs).

**TABLE 2:** Breeding stocks and their expected genetic contribution in breeding program.

High yield potential	High quality potential	Pest tolerance/resistance	Drought tolerance	High soil pH tolerance	Cold tolerance	Genetic study
TRFK 31/8	TRFK 6/8^9^	TRFK 7/9^3^	TRFCA SFS150	EPK TN14-3	EPK TN14-3	TRFK 12/2^1^
TRFK 303/577^8^	GW Ejulu-L	TRFK 57/15^3^	TRFK 303/577^8^	NDT Tai	TRFCA SFS150	TRFK K-Purple
TRFK 301/4	EPK TN 15-23	AHP SC31/37^3^			EPK C12	TRFK 31/30^2^
TRFK 301/5		AHP S15/10^3^			NRIT Yabukita^6^	TRFK 311/287^2^
EPK C12		EPK TN14-3^5^			NRIT Yutakamidori^6^	TRFK 382/1^7^
BBLK 35		TRFK 303/1199^3^				TRFK 382/2^7^
AHP S15/10^9^		TRFK 54/40^4^				TRFK 386/2^7^
AHP SC12/28^9^		TRFCA SFS150^3^				TRFK 371/1^7^
AHP SC31/37		AHP CG28U864^4^				TRFK 306^10^
AHP CG28V929^9^		TRFK 301/1^4^				Wild *Camellia* spp.
AHP CG28U864		TRFK L/16^4^				

*^1^Non fermenter; ^2^tetraploid; ^3^resistant to red crevice mite ^4^susceptible to scales; ^5^preferred but highly tolerant to red crevice mite; ^6^green tea varieties-low catechin content; ^7^triploid; ^8^susceptible to root knot nematodes; ^9^very susceptible to water stress; ^10^anthocyanin tea cultivar.*

Attempts to improve stress tolerance in tea through conventional breeding programs have, however, met limited success, partially attributed to the robust breeding programs and improved crop husbandry (Kamunya et al., 2010, 2012). However, due to the lack of sufficient genetic information about genes that govern this complex trait and its component secondary traits, progress in tea improvement has been slow. Research has shown that DT varies considerably between tea cultivars (Ng’etich et al., 2001; Cheruiyot et al., 2008; Carr, 2010a, b; Kamunya et al., 2010), which further suggests the need for investigating the genetic architecture and adaptive responses of tea to drought. Limitations in conventional breeding coupled with advances in molecular breeding have unveiled a new era in tea breeding.

### From Conventional to Molecular Breeding

Understanding the genetics of how organisms adapt to changing environment is crucial for the adaptability of a genotype (Chinnusamy and Zhu, 2009). Due to the limitations associated with conventional breeding approaches, other means of genetic improvement are being explored. Availability of molecular tools arising from the development of molecular markers manifested a significant advancement in crop improvement in the 1980s. Different marker systems such as randomly amplified polymorphic DNA (RAPD), restriction fragment length polymorphism (RFLP), amplified fragment length polymorphism (AFLP), sequence tagged sites (STS), single-strand conformation polymorphism (SSCP), inter simple sequence repeat (ISSR), simple sequence repeat (SSR) or microsatellite, Diversity Arrays Technology (DArT) microarray and chloroplast DNA (cpDNA) have been developed and applied in tea breeding (Wachira et al., 2001; Mondal et al., 2004; Chen et al., 2007; Sharma et al., 2009; Wambulwa et al., 2016a, b, 2017; Koech et al., 2018, 2019). These markers have been applied in genetic studies relating to assessment of genetic diversity and germplasm characterization, genotype identification and fingerprinting, estimation of genetic distances between populations, assessment of mating systems, detection of quantitative trait loci (QTLs), and marker-assisted selection (MAS) in tea (Wachira et al., 1995, 1997; Paul et al., 1997; Hackett et al., 2000; Muoki et al., 2007; Kamunya et al., 2010; Koech et al., 2018, 2019).

Most attributes of agricultural importance frequently manipulated by plant breeders (e.g., size, shape, yield, quality, tolerance to abiotic, and sometimes biotic stresses) display a quantitative mode of inheritance and normally exhibit continuous variation (Collard et al., 2005). Continuous variation in a phenotype can be explained by the independent actions of many distinct genetic factors, each having small effects on the overall phenotype. Detection of QTL controlling complex traits followed by selection has become a common approach for selection in crop plants. QTLs or linkage mapping aims at identifying genomic regions that could be useful to analyze genetics of complex traits (Stapley et al., 2010). QTLs are typically mapped by crossing parental varieties contrasting for the trait of interest to generate a mapping population which are then scored for phenotypes and genotyped so as to identify the parts of the genome that improve the trait and the genome regions that influence component trait linked to the main trait. Once achieved, targeting of genomic regions for varietal improvement could be possible through MAS, thereby shortening the development and release of elite varieties for commercialization (Hackett et al., 2000; Kamunya et al., 2010). The approach is helpful in tea where conventional breeding technique takes over 20 years to develop an improved cultivar. Integration of molecular markers in breeding and clonal selection would also help in reducing the number of clones/seedlings for field testing (Kamunya et al., 2010).

The first genetic linkage maps for tea was constructed using RAPD and AFLP markers and covered 1349.7 cM with an average distance of 11.7 cM (Hackett et al., 2000). In addition, QTLs for yield, DT, quality traits [percent total polyphenols (%TP)], fermentability (FERM), theaflavins (TF), thearubigins (TR), and pubescence (PUB) has been reported (Kamunya et al., 2010). Here, bulk segregant analysis followed by complete genotyping identified 260 RAPD and AFLP informative markers. Of these, 100 markers showing 1:1 segregation, were used to generate a linkage map with 30 (19 maternal and 11 paternal) linkage groups spanning 1411.5 cM with mean interval of 14.1 cM between loci. On the basis of the map, QTL analysis was done on data over two sites. A total of 64 putative QTLs for various traits across different sites were detected. More recently, phenotypic data for two segregating tea populations was used to identify QTL influencing tea biochemical and drought stress traits based on a consensus genetic map constructed using the DArTseq platform (Koech et al., 2018). The map consisted of 15 linkage groups from the two populations comprised 261 F1 clonal progeny and spanned 1260.1 cM with a mean interval of 1.1 cM between markers. Both interval and multiple QTL mapping revealed a total of 47 putative QTL in the 15 LGs associated with tea quality and percent RWC at a significant genome-wide threshold. These markers contribute greatly to adoption of MAS for DT and tea quality improvement. However, positional cloning of genes controlling important traits in tree species is difficult (Stirling et al., 2001), partly due to the complexity of gene networks and interactions among or between genetic elements and the environment (Ribaut and Ragot, 2007). Such limitations can be overcome by adopting new approaches that exclude the need to map QTLs.

## Future Prospects

Great progress has been made in assessment of the relationship between tea productivity and climate change. In order to anticipate the effects of climate change on tea and provide scientists with necessary knowledge and tools, multidisciplinary approaches should be embraced. The approaches outlined below are recommended:

(a) It would be important to quantify the long term response of the tea plant to elevated CO_2_ concentrations so as to understand the link between carbon supply and plant growth. The extensive use of artificial environments such as the free air CO_2_ enrichment (FACE) technology can help examine the magnitude of elevated CO_2_ on tea yield and quality at the level of the ecosystem.
(b) Invest in alternative breeding approaches such as mutation breeding for increased genetic variability. This should be followed by standardizing selection procedures which attempt to identify useful genotypes.
(c) Studies have shown that the response of plants to a combination of stresses is unique and cannot be directly extrapolated from the response of plant to each of the different stresses applied individually. Further, simultaneous occurrence of several stresses enhances the intensity of lethality to crop as compared to that imposed by a single stress. Nevertheless, little is known about the molecular mechanisms underlying the acclimation of tea to a combination of different stresses. Systems biology approach facilitate a multi-targeted approach for understanding complex molecular regulatory networks associated with stress adaptation and tolerance. The approach can help overcome limitations associated with morphological, biochemical and molecular adaptation of the plants to stress. Tolerance to a combination of different stress conditions, particularly those that mimic the field environment, should be the focus of future research programs aimed at developing improved varieties and plants with enhanced tolerance to naturally occurring environmental conditions.
(d) Establish multi-stakeholders collaborations aimed at developing sustainable adaptation strategies for management of climate risks associated with climate change in the tea industry.

## Author Contributions

All authors wrote and revised the manuscript.

## Conflict of Interest

The authors declare that the research was conducted in the absence of any commercial or financial relationships that could be construed as a potential conflict of interest.
